# Using recursive feature elimination in random forest to account for correlated variables in high dimensional data

**DOI:** 10.1186/s12863-018-0633-8

**Published:** 2018-09-17

**Authors:** Burcu F. Darst, Kristen C. Malecki, Corinne D. Engelman

**Affiliations:** Department of Population Health Sciences, School of Medicine and Public Health, University of Wisconsin, 610 Walnut Street, 1007 WARF, Madison, WI 53726 USA

**Keywords:** Genomics, Genetics, Epigenomics, Methylation, Machine-learning, Omics, Integration, High-dimensional data, Random forest, Recursive feature elimination, Correlation

## Abstract

**Background:**

Random forest (RF) is a machine-learning method that generally works well with high-dimensional problems and allows for nonlinear relationships between predictors; however, the presence of correlated predictors has been shown to impact its ability to identify strong predictors. The Random Forest-Recursive Feature Elimination algorithm (RF-RFE) mitigates this problem in smaller data sets, but this approach has not been tested in high-dimensional omics data sets.

**Results:**

We integrated 202,919 genotypes and 153,422 methylation sites in 680 individuals, and compared the abilities of RF and RF-RFE to detect simulated causal associations, which included simulated genotype–methylation interactions, between these variables and triglyceride levels. Results show that RF was able to identify strong causal variables with a few highly correlated variables, but it did not detect other causal variables.

**Conclusions:**

Although RF-RFE decreased the importance of correlated variables, in the presence of many correlated variables, it also decreased the importance of causal variables, making both hard to detect. These findings suggest that RF-RFE may not scale to high-dimensional data.

## Background

Although recent improvements in high-throughput technology enable the collection of large omics data sets for many biological fields, analysis methods to handle these data are still in their infancy. The variety of currently available omics data types provides the opportunity to move toward a systems biology approach, which is essential to understand the genomic complexities of non-Mendelian traits. The alleged “missing heritability” of complex traits is likely, in part, the result of most studies focusing on linear models within single data types, thereby limiting findings to variants that are independently correlated with disease [1]. However, genetic variants likely interact with each other and other biologic processes in complex nonlinear ways to influence disease. Integrating multiple omics data types is thought to be a powerful approach, allowing for more thorough and comprehensive modeling of complex traits [2, 3]. Only recently have researchers begun tackling the complexities and analytic challenges that omics integration poses and thus, gold standards do not yet exist.

Random forest (RF) is a machine-learning method that may be a good candidate for integrating omics data as it generally works well with high-dimensional problems and can identify strong predictors of a specified outcome without making assumptions about an underlying model [4]. However, a common problem of high-dimensional data sets is the presence of correlated predictors, which impact RF’s ability to identify the strongest predictors by decreasing the estimated importance scores of correlated variables [5]. A suggested solution is the Random-Forest-Recursive Feature Elimination (RF-RFE) algorithm [5]. RFE was initially proposed to enable support vector machines to perform feature selection by iteratively training a model, ranking features, and then removing the lowest ranking features [6]. This method has been similarly applied to RF [7, 8] and found to be beneficial in the presence of correlated features [5].

In this study, we assessed how well RF-RFE mitigates the effects of correlated variables in high-dimensional integrated omics data by comparing the ability of RF-RFE to RF without RFE to detect simulated associations, including interactions, in the presence of correlated variables. Data were based on the Genetics of Lipid Lowering Drugs and Diet Network study [9] and included genomic, epigenomic, and triglyceride (TG) data provided for GAW20. Analyses were conducted with knowledge of the simulated model.

## Methods

### Data set

The data set provided by GAW20 organizers included a simulated pharmacoepigenetic effect of a fictitious drug on TG response, where major effects include interactions that depend on an individual’s genotype and corresponding methylation state. Specifically, there are five simulated causal single nucleotide polymorphisms (SNPs) that express their influence on TG response to treatment when their five corresponding nearby cytosine-phosphate-guanine (CpG) sites are sufficiently unmethylated posttreatment. This analysis used genome-wide genotypes, simulated posttreatment genome-wide methylation, and TGs measured on two consecutive days pretreatment and simulated on two consecutive days posttreatment, for a total of four TG measures.

Consistent with the simulation model, we calculated TG response by subtracting the average log pretreatment from the average log posttreatment TG measures and then adjusted this difference by baseline TG levels using linear regression. The resulting residuals were used as the outcome; SNPs and posttreatment CpG sites were predictors in RF.

A total of 680 participants had all three data types and were included in the analyses. Because of the computational demands of the analyses, we focused on chromosomes 1, 6, 8, 10, and 17 (202,919 SNPs and 153,422 simulated posttreatment CpGs, for a total of 356,341 variables), which contained the five causal SNPs and their corresponding methylation sites. Furthermore, we used the 84th simulation replicate, which was suggested by the GAW20 organizers to be most representative of the 200 simulations provided. Correlation between predictors was calculated using Pearson r^2^. Regional association plots displaying results from both RF and RF-RFE were created using LocusZoom v1.3 [10].

### Random forest

RF is a machine-learning algorithm that ranks the importance of each predictor included in a model by constructing a multitude of decision trees [4]. Each node of a tree considers a different subset of randomly selected predictors, of which the best predictor is selected and split on. The criterion used to determine the best predictor was decreased in node impurity, measured with the estimated response variance, which is the default method used for regression trees in the ranger implementation of RF that was used in this study [11]. Each tree is built using a different random bootstrap sample, which consists of approximately two-thirds of the total observations and is used as a training set to predict the data in the remaining out-of-bag (OOB) sample, or testing set. Predictions for each variable are aggregated across all trees and the mean square error (MSE) of the OOB estimates is calculated. The MSE_OOB_ and percentage of variance explained are used to evaluate the performance of each RF.

### Recursive feature elimination

To assess whether RF-RFE improved upon RF alone, we assessed the importance scores attained after running RF once and after running it recursively, using the initial RF as the first of the recursive runs in the RF-RFE approach. The RF-RFE approach consisted of (a) running RF to determine initial importance scores, (b) removing the bottom 3% of variables with the lowest importance scores from the data set (3% was chosen because of the high computational demands of using a lower threshold; this resulted in a total of 324 RF runs), and (c) assigning ranks to removed variables according to the order in which they were removed and their most recent importance scores (ie, importance scores are only compared within runs, not between runs). This was performed iteratively using the reduced data set created in step two until 3% of the number of remaining variables rounds to zero (ie, no further variables could be removed).

### Parameter tuning and model runtimes

In RF, the number of predictors sampled for splitting at each node, mtry, and the number of trees in the forest are the two primary tuning parameters [12]. For this analysis, 8000 trees were used. When the majority of features are noise or a very large number of features are being used, an mtry of 0.1*p, where p is the number of predictors in the model, has been suggested to be a more appropriate choice than the default mtry =$\sqrt{\mathrm{p}}$ [13, 14]. Thus, when p > 80 and likely still contained many noisy variables, we used an mtry of 0.1*p, and after features were recursively removed from the model and p ≤ 80, we used the default mtry. These parameters produced reasonably low MSE_OOB_s when compared to others. Permutation variable importance mode was used.

The initial RF took approximately 6 h and the RF-RFE took approximately 148 h to run on a Linux server with 16 cores and 320GB of RAM.

## Results

None of the causal CpG sites were highly correlated with any other variable (all r^2^ < 0.04), and causal SNPs were only highly correlated with other SNPs, indicating that linkage disequilibrium was likely the strongest contributor to correlation in this data set. Table 1 provides summary statistics for the causal SNPs and CpGs, including minor allele frequencies (MAFs) for SNPs and means for CpGs, simulated effects based on the full 200 simulated replicates, and effects tested with linear regression models using the 84th simulated replicate used for this study. These models confirm that the causal SNPs have main effects, but causal CpGs only have effects through interactions with their corresponding SNPs.

**Table 1:** Summary statistics and variable importance rankings of simulated causal effects on TG

						RF*	RF-RFE
					r^2^	−0.00203	0.19217^a^
					MSE_OOB_	0.07378	0.05948^a^
Chr	Causal SNP/CpG	MAF or Mean (SD)	Simulated h^2†^	Main Effects, β(SE), *p* value	Interaction Effects, β(SE), *p* value	Rank (percentile rank)	
1	rs9661059	0.12	0.125	.14 (0.02), **<0.0001**	−0.19 (0.07), **0.0109**	1 (100.0)	20 (100.0)
	cg00000363	0.49 (0.33)	–	−0.05 (0.03), 0.0873		8680 (97.6)	239,755 (32.7)
6	rs736004	0.09	0.075	0.09 (0.03), **0.0005**	−0.30 (0.08), **0.0001**	13,480 (96.2)	766 (99.8)
	cg10480950	0.54 (0.33)	–	−0.04 (0.03), 0.1711		5332 (98.5)	232,579 (34.7)
8	rs1012116	0.20	0.100	0.08 (0.02), **<0.0001**	−0.21 (0.06), **0.0002**	50,218 (85.9)	333,504 (6.4)
	cg18772399	0.56 (0.33)	–	−0.002 (0.03), 0.9466		339,475 (4.7)	301,855 (15.3)
10	rs10828412	0.14	0.025	0.07 (0.02), **0.0007**	−0.07 (0.07), 0.2770	2984 (99.2)	330,516 (7.3)
	cg00045910	0.49 (0.34)	–	0.01 (0.03), 0.7176		263,465 (26.1)	231,315 (35.1)
17	rs4399565	0.41	0.050	0.04 (0.02), **0.0142**	−0.13 (0.05), **0.0038**	11,078 (96.9)	196,276 (44.9)
	cg01242676	0.46 (0.32)	–	0.01 (0.03), 0.8159		350,420 (1.9)	350,420 (1.7)

Bolded values are significant at a *p*<0.05

*RF is the first RF in RF-RFE

^a^r^2^ and MSE_OOB_ are averaged over all 324 RFs in the RF-RFE column

^†^Simulated h^2^ was provided by GAW20 organizers and based on full 200 simulations; main and interaction effects are calculated within the data set used for this study, which uses the 84th simulation replicate. Effects are calculated with linear regression models using the residual of change in TG after adjusting for baseline TG as the outcome. Interaction effects include the main effects of the interaction terms being tested in the given model

Abbreviations: β Effect size, *Chr* Chromosome, h^2^ Heritability

**Fig. 1:**
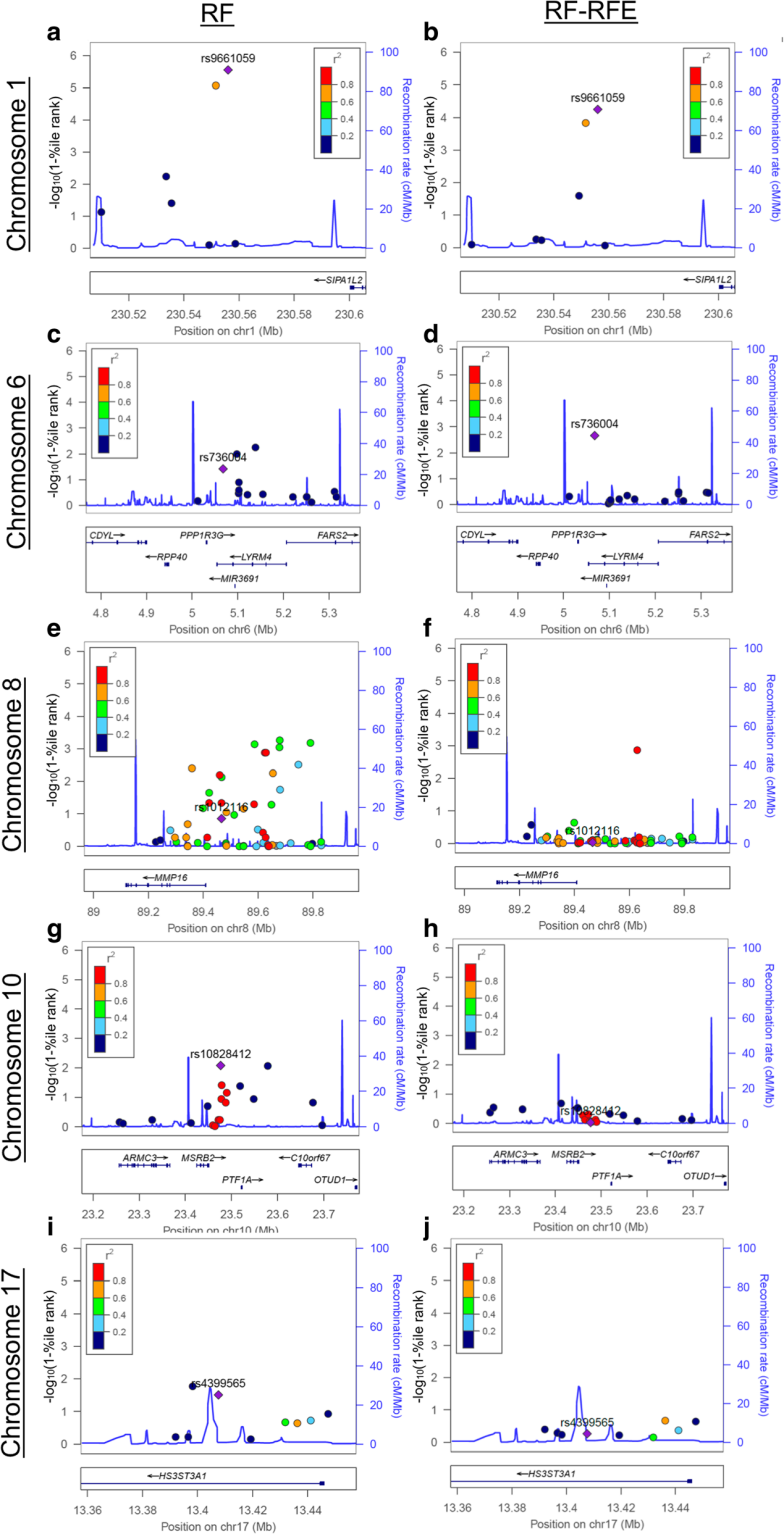
Regional association plots showing RF and RF-RFE importance rankings of causal and correlated SNPs (r^2^ > 0.10). The causal SNP in each plot is shown by the purple diamond, with the reference SNP number indicated above. A higher value on the y-axis indicates a higher importance score and better rank. **a**. RF importance rankings for chromosome 1. **b**. RF-RFE importance rankings for chromosome 1. **c**. RF importance rankings for chromosome 6. **d**. RF-RFE importance rankings for chromosome 6. **e**. RF importance rankings for chromosome 8. **f**. RF-RFE importance rankings for chromosome 8. **g**. RF importance rankings for chromosome 10. **h**. RF-RFE importance rankings for chromosome 10. **i**. RF importance rankings for chromosome 17. i. RF-RFE importance rankings for chromosome 17

Table 1 provides RF and RF-RFE rankings based on importance scores for the five simulated causal SNPs and CpGs. Figure 1 visually shows the rankings of causal SNPs and their correlated SNPs with an r^2^ > 0.10. It was not uncommon for correlated SNPs to be ranked similarly to causal SNPs by RF and this similarity was not always influenced by the strength of correlation (Fig. 1c, e and g).

RF was generally able to identify causal SNPs fairly well. Despite being simulated to have the second highest effect size, rs1012116 in chromosome 8 was the lowest ranking simulated SNP in RF, but was still in the 85.9 percentile (see Table 1). Of interest, this SNP had the greatest number of highly correlated SNPs when compared to the four other causal SNPs (see Fig. 1e). The causal SNP with the largest effect size and the fewest correlated SNPs, rs9661059 in chromosome 1, was impressively ranked as the single top predictor in RF of the total 356,341 variables included (see Table 1 and Fig. 1a), while a highly correlated SNP (rs9725734, r^2^ = 0.84) was ranked third (orange marker in Fig. 1a). RF was able to identify causal CpG sites cg00000363 and cg10480950 (each ranked >97th percentile), both of which were in chromosomes that did not have many highly correlated SNPs (chromosomes 1 and 6, respectively) (Table 1 shows rankings and Fig. 1a and c show SNP correlations). When tested in linear regression models, these two CpG sites also had the smallest *p* values compared to the remaining causal CpGs, which RF ranked very poorly (see Table 1).

Figure 1 shows that rankings of the correlated SNPs notably decreased in RF-RFE when compared to RF. The top ranking SNP in RF (causal rs9661059 in chromosome 1) ranked slightly lower in RF-RFE (rank = 20) (see Table 1 and Fig. 1a and b), as did the SNP it was highly correlated with (rs9725734, rank = 52) (orange marker in Fig. 1a and b). The causal SNP in chromosome 6, rs736004, ranked higher in RF-RFE than in RF (99.8 percentile) (see Table 1 and Fig. 1c and d). Neither of these SNPs had many highly correlated variables. However, in the presence of many highly correlated variables, rankings of the causal SNPs greatly decreased in RF-RFE when compared to RF alone. This was true in chromosomes 8 and 10, where both causal SNPs were ranked in the 10th percentile by RF-RFE (see Table 1 and Fig. 1e, f, g and h). Both of these causal SNPs had many highly correlated variables. The causal SNP in chromosome 17, rs4399565, was not correlated with many other SNPs, but a few of these were moderately correlated with rs4399565 (see Fig. 1i). This SNP had a much lower ranking in RF-RFE (44.9 percentile) (see Table 1 and Fig. 1j), but not quite as low as those in chromosomes 8 and 10. All CpG sites ranked very poorly in RF-RFE, with the highest percentile rank being 35.1 (see Table 1).

Background SNPs simulated by the GAW20 organizers to have small effect sizes on TG (all heritabilities = 0.001) were generally not ranked highly by RF and did not change rankings greatly with RF-RFE (data not shown). Of the 25 background SNPs present in our data, RF ranked 19 and RF-RFE ranked 16 of them below the 70th percentile.

As a consequence of the random nature of RF, RF and RF-RFE were run a second time and results were generally consistent, particularly the strongest findings.

## Discussion

In this study, we used simulated data to assess the ability of RF-RFE to ameliorate the effects that correlated variables have on RF importance scores using high-dimensional integrated omics data. Comparing importance score rankings from RF and RF-RFE, we found that RF-RFE ranked SNPs that were correlated with causal SNPs lower than RF ranked them, as anticipated. Causal SNPs with fairly simple correlation structures that were not highly correlated with many other SNPs (ie, those within chromosomes 1 and 6) received similar or higher importance score rankings in RF-RFE than in RF, making them easier to distinguish from the correlated noncausal SNPs. However, when many SNPs were highly correlated with the causal SNP, RF-RFE ranked causal SNPs very poorly. Thus, RF-RFE may not be an effective method to use in data sets that contain many highly correlated variables. However, analyses with a larger number of simulated variables with strong effects would be more conclusive regarding the influence of correlation on RF-RFE with omics data.

RF identified causal variables more strongly when they had fewer correlated variables, further supporting the reported influence of correlated variables on importance scores [15]. Even with this limitation, RF ranked most of the simulated causal variables highly, confirming that it is a strong approach for variable selection in high-dimensional data. It may not be a strong approach for detecting subtle effects though, as was indicated by its inability to detect simulated background SNPs with small effect sizes.

Although RF-RFE may not ameliorate the effects of correlated variables in the presence of many highly correlated variables, it could be an effective method for nongenetic omics data sets. Because of linkage disequilibrium, genomic data typically include many highly correlated variants, but this correlation structure is unlikely to be present in other omics data types. While our analyses did include epigenomic data, CpGs were not simulated to have main effects, as they only had interaction effects through corresponding SNPs. Interactions are particularly difficult to assess in RF when the interacting features lack main effects as they are unlikely to be selected and split on at all [16], which suggests that they are unlikely to rank highly in RF, and could explain why RF-RFE did not improve their rankings. Thus, we were unable to explicitly assess the performance of RF-RFE with this nongenetics omics data set.

Despite RF being well suited to handle nonlinear effects, without performing additional analyses, importance scores alone do not provide information about which variables may be interacting. Millions of pairwise possibilities would have to be further tested based on the current results to identify the simulated interaction between the two causal CpG sites in chromosomes 1 and 6 that ranked well in RF and their corresponding SNPs. Even then, power issues would likely make these interactions impossible to detect using traditional methods. The other three causal CpG sites ranked very poorly in RF and did not suggest interactions. However, this was not unexpected as permutation importance scores were not designed to detect interactions [16] and reportedly fail to do so in high-dimensional data with weak marginal effects [17]. Although it has been shown that RF is influenced by interactions, it is very difficult to specifically identify which variables are interacting with current variable importance methods [16].

## Conclusions

To conclude, RF-RFE may not be an appropriate method when many highly correlated features are present in high-dimensional omics data. Further research is needed to assess its effectiveness in nongenetic omics data. Although correlated variables impact the performance of RF, RF detected simulated associations more strongly than RF-RFE and is a robust method for high-dimensional data.

## Abbreviations

CpG: Cytosine-phosphate-guanine
GAW: Genetic Analysis Workshop
MAF: Minor allele frequency
MSE: Mean square error
MSE_OOB_: Mean square error of out of bag estimates
OOB: Out-of-bag
RF: Random forest
RFE: Recursive feature elimination
RF-RFE: Random forest-recursive feature elimination
SNP: Single nucleotide polymorphism
TG: Triglyceride
